# Diagnosis and treatment of congenital tricuspid valve malformation in a case of monozygotic twins

**DOI:** 10.1186/s13019-022-01911-w

**Published:** 2022-07-15

**Authors:** Pin Shen, Qin Xie, Runwei Ma, Yunxing Dong, Qiang Wang, Yi Sun

**Affiliations:** 1 Department of Cardiovascular Surgery, Fuwai Yunnan Cardiovascular Hospital, 528, Shahe North Road, Wuhua District, Kunming, 650000 Yunnan People’s Republic of China; 2 Yunnan Key Laboratory of Stem Cell and Regenerative Medicine, Biomedical Engineering Research Center, Kunming Medical University, Kunming, 650500 Yunnan People’s Republic of China; 3 Department of Vascular Surgery, The Second People’s Hospital of Yunnan Province, Kunming, 650500 Yunnan People’s Republic of China

**Keywords:** Monozygotic twins, Congenital heart disease, Ebstein’s anomaly

## Abstract

**Background:**

Congenital tricuspid valve malformations are known to occur, but tricuspid valve malformations associated with twins are rarely reported. We report this case from the point of view of a medical history, an auxiliary examination and a genetic pathogenesis to provide a reference for our peers.

**Case presentation:**

We report a rare case of congenital heart disease in monozygotic twins of Hui nationality in Yunnan-Guizhou Plateau, they are normal conception. Twin 1 had Ebstein’s anomaly, and received surgical treatment and recovered satisfactorily. Twin 2 had only partial tricuspid septal prolapse, and pulmonary hypertension occurred during follow-up.

**Conclusions:**

It is necessary to carry out individualized diagnosis and treatment for twins and follow-up observation by echocardiography for a long time. Choosing the right time for cardiac surgery is of great significance to the treatment of the disease.

## Background

CHD is more common in twins than in singletons, but the overall survival prognosis of CHD in human MZ twins is similar to that in singletons [1]. EA is a congenital tricuspid malformation, usually with a tricuspid septal valve, posterior valve downward movement, and valvular abnormality. The anterior valve usually has a sail-shaped dilatation, and the specific embryological mechanism is not clear. The individual treatment of children with EA varies greatly, and the standard of surgical intervention is also controversial. Critically ill children with severe cyanosis and metabolic acidosis in infancy may need early surgical intervention, while patients with asymptomatic or mild symptoms may only need observation [2]. Patients with obvious tricuspid regurgitation with symptoms or heart failure should also receive surgical intervention to avoid the increase in morbidity and mortality caused by delayed intervention. Tricuspid valve repair is usually preferable to replacement where technically feasible. Radiofrequency ablation should be performed when severe arrhythmia is present [3].

## Case introduction

**Fig. 1:**
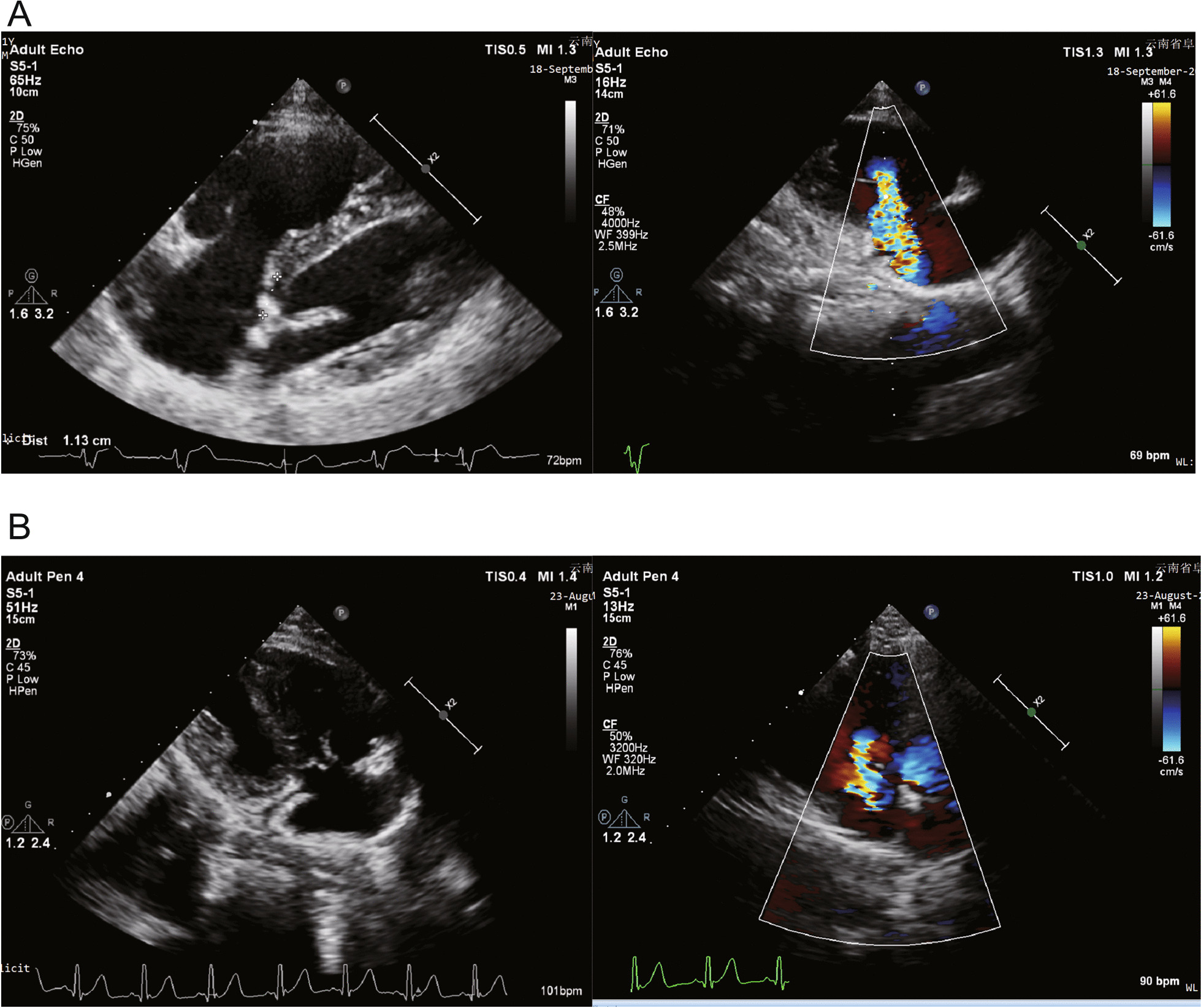
**a** The oblique four-chamber section of transthoracic echocardiography of Twin 1 shows that the septal lobe of the tricuspid valve moves downward to the apical part of the heart by transthoracic echocardiography, and the right ventricle inflow tract section by transthoracic echocardiography shows a large volume of tricuspid regurgitation by colour Doppler. **b** The oblique four-chamber section by transthoracic echocardiography of Twin 2 shows that the septal lobe of the tricuspid valve protrudes slightly into the right atrium during systole; the colour Doppler shows tricuspid regurgitation in the inflow section of the right ventricle by transthoracic echocardiography

**Fig. 2:**
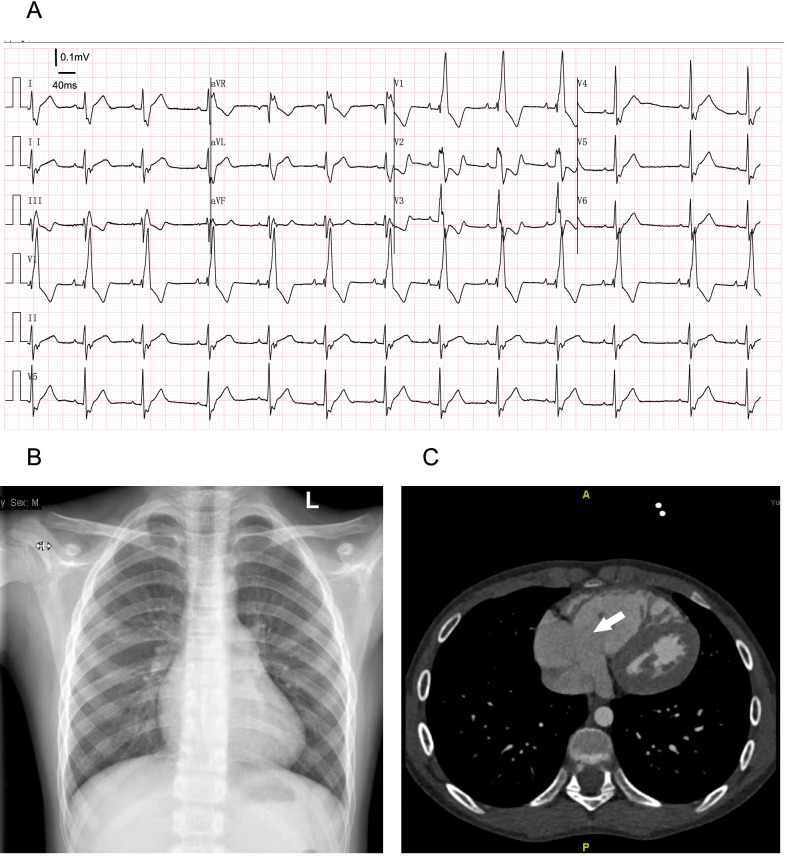
**a** The electrocardiogram of Twin 1 shows a complete right bundle branch block. **b** the Chest X-ray of Twin 1 shows an enlarged right atrium, a clear lung field, and a cardiothoracic ratio of 0.5. **c** Twin 1’s CT scan, with an arrow showing tricuspid annulus enlargement

Twin 1 was an 11-year-old male with a height of 145 cm and weight of 36 kg. The patient was born prematurely at 36 weeks of gestation. On the 40th day after birth, the patient underwent routine physical examination, had a heart murmur during auscultation, and was diagnosed with congenital moderate tricuspid regurgitation by ultrasound in the local hospital. The hospital advised the patient's family members to continue to observe the condition. The patient was often prone to colds in infancy. Recently, the patient developed asthma after exercise, and his physical activity was slightly limited, so he went to Fuwai Yunnan Cardiovascular Hospital. A physical examination showed that a soft systolic murmur of grade 2 at beat 6 could be heard in the fourth intercostal space of the left margin of the sternum. The echocardiographic report was as follows: EA, type A according to the Carpentier classification, with right atrium enlargement, a TAPSE of 20 mm, tricuspid annulus enlargement, and an internal diameter up to 38 mm. The anterior tricuspid leaflet was long, the septal lobe moved downward approximately 12 mm, the abnormal chordae tendineae were attached to the right ventricular wall, the valvular lobe was poorly closed, and there was a large volume of tricuspid regurgitation (Fig. 1a). An electrocardiogram showed arrhythmias and complete right bundle branch block (Fig. 2a). The chest X-ray showed enlargement of the right atrium (Fig. 2b). CT showed tricuspid enlargement (Fig. 2c). Surgical intervention was performed under general anaesthesia and cardiopulmonary bypass. During the operation, the diameter ratio of the aorta to the pulmonary artery was approximately 1:1. A two-needle 5–0 double needle belt gasket was used to contract the tricuspid valve with a DeVega ring and to fold part of the atrialized right ventricle to correct three kinds of valve downward deformities. The patient’s chest was closed successfully and he was sent to the intensive care unit. The time of cardiopulmonary bypass (62 min) was 17 h after the operation in the intensive care unit, and 7 days after treatment, he was discharged safely. The patient was followed up for 2 years after the operation, and the prognosis was good.

Twin 2 was also examined. The history of Twin 2 was similar to that of Twin 1, but there were no clinical symptoms. The ultrasonic electrocardiogram of Twin 2 showed that the tricuspid septal lobe was partially prolapsed, the right atrium was enlarged, the tricuspid valve leaf was slightly thickened, the tricuspid septal valve tip adhered to the interventricular septum, and the activity of the tricuspid septum was slightly limited. Part of the valve body near the posterior part of the tricuspid septal lobe slightly protruded into the right atrium during systole, resulting in valve opening, poor closure and moderate tricuspid regurgitation (Fig. 1b). The patient did not meet the indication for surgical treatment, but we still instructed the patient to follow up regularly, and the patient has been followed up for 2 years. Unfortunately, the most recent echocardiogram estimated mild pulmonary hypertension, but the patient refused to undergo further examination by right cardiac catheterization. We asked the patient to limit exercise, take oral diuretics, reduce heart load, and continue close follow-up.

## Discussion

EA is a rare CHD, accounting for less than 1% of all CHDs. It was first described by Wilhelm Ebstein in a report published in 1866 [4]. EA is even more rare in twins; a single fertilized egg divides into twins, who share a placenta, and there is a large amount of vascular communication between twins. When blood exchange is not equal, twin transfusion syndrome can cause changes in foetal cardiac load, but the severity of tricuspid regurgitation is still unclear [5]. MZ twins are thought to be genetically identical, but inconsistent phenotypes have been found in MZ twins. Differences in chromosome structure or gene mutations are one of the reasons for these inconsistent phenotypes. Other mechanisms leading to CHD differences in MZ twins include environmental factors, epigenetic changes, noncoding DNA mutations or oligogenic mechanisms [6]. In addition to epigenetics, the influence of the local placenta is also considered to be an important factor in the difference in CHD in MZ twins. The vascular connection between the placenta and the insertion site of the umbilical cord leads to blood flow imbalance, resulting in relatively insufficient perfusion in MZ twins [7].

Most patients with EA have normal physical development, and ultrasonic electrocardiogram can diagnose this kind of disease [8, 9]. In the past, patients with severe EA often underwent tricuspid valve replacement, but the clinical results were not satisfactory. Modified tricuspid valvuloplasty greatly reduced the mortality of patients with EA. A 20-year follow-up showed a survival rate of 82% [10]. The MZ twins were in stable condition for 12 years after birth until Twin 1 showed obvious symptoms of fatigue, arrhythmia and tricuspid annulus enlargement, so the surgical indications were clear, and annuloplasty was performed with a DeVega ring [11].

## Conclusion

In this case of MZ twins, the changes in the tricuspid valve were different. We took effective treatment measures for the twins and followed up for 2 years. Twin 1 underwent surgical intervention with a good prognosis, and no adverse events have been reported thus far. Twin 2 still needs further observation. In the future, individualized treatment for such patients is desirable; of course, this kind of disease is relatively less common and physicians lack experience; it is hoped that more cases are reported, and evidence-based treatments are obtained so that patients have better benefits.

## Data Availability

Not applicable.

## Abbreviations

CHD: Congenital heart disease
MZ: Monozygotic
EA: Ebstein’s anomaly
